# Evolution and assembly of *Anopheles aquasalis*'s immune genes: primary malaria vector of coastal Central and South America and the Caribbean Islands

**DOI:** 10.1098/rsob.230061

**Published:** 2023-07-12

**Authors:** Cesar Camilo Prado Sepulveda, Rodrigo Maciel Alencar, Rosa Amélia Santana, Igor Belém de Souza, Gigliola Mayra Ayres D'Elia, Raquel Soares Maia Godoy, Ana Paula Duarte, Stefanie Costa Pinto Lopes, Marcus Vinicius Guimarães de Lacerda, Wuelton Marcelo Monteiro, Rafael Nacif-Pimenta, Nágila Francinete Costa Secundino, Leonardo Barbosa Koerich, Paulo Filemon Paolucci Pimenta

**Affiliations:** ^1^ Fundação de Medicina Tropical Dr. Heitor Vieira Dourado, Manaus, Amazonas, Brazil; ^2^ Programa de Pós-Graduação em Medicina Tropical, Fundação de Medicina Tropical Heitor Vieira Dourado, Universidade do Estado do Amazonas, Manaus, Amazonas, Brazil; ^3^ Instituto de Pesquisas René Rachou, Fundação Oswaldo Cruz, Belo Horizonte, Minas Gerais, Brazil; ^4^ Programa de Pós-Graduação em Ciências da Saúde, FIOCRUZ – Belo Horizonte. Minas Gerais, Brazil; ^5^ Instituto de Pesquisas Leônidas e Maria Deane, Fundação Oswaldo Cruz, Manaus, Amazonas, Brazil; ^6^ University of Texas Medical Branch, Galveston, TX, USA; ^7^ Departament of Epidemiology of Microbial Disease, Yale School of Public Health, New Haven, CT, USA; ^8^ Departamento de Parasitologia, Universidade Federal de Minas Gerais, Belo Horizonte, Minas Gerais, Brazil

**Keywords:** anophelines, diverged evolution, genome, immune response, microbiota

## Abstract

Anophelines are vectors of malaria, the deadliest disease worldwide transmitted by mosquitoes. The availability of genomic data from various *Anopheles* species allowed evolutionary comparisons of the immune response genes in search of alternative vector control of the malarial parasites. Now, with the *Anopheles aquasalis* genome, it was possible to obtain more information about the evolution of the immune response genes. *Anopheles aquasalis* has 278 immune genes in 24 families or groups. Comparatively, the American anophelines possess fewer genes than *Anopheles gambiae* *s.* *s*., the most dangerous African vector. The most remarkable differences were found in the pathogen recognition and modulation families like FREPs, CLIP and C-type lectins. Even so, genes related to the modulation of the expression of effectors in response to pathogens and gene families that control the production of reactive oxygen species were more conserved. Overall, the results show a variable pattern of evolution in the immune response genes in the anopheline species. Environmental factors, such as exposure to different pathogens and differences in the microbiota composition, could shape the expression of this group of genes. The results presented here will contribute to a better knowledge of the Neotropical vector and open opportunities for malaria control in the endemic-affected areas of the New World.

## Graphical abstract

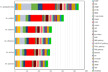

## 1. Introduction

Malaria is a severe public health problem in several tropical and subtropical areas. It is caused by parasites of the genus *Plasmodium,* and is of most concern in the African, Asian and American continents. Annually there are 229 million cases that result in nearly half a million deaths worldwide, affecting mainly pregnant women and children [1]. The disease is transmitted to humans by the bite of female *Anopheles* sp. Among more than 400 known species of *Anopheles*, 41 are recognized vectors worldwide, 9 of which are found in the Americas, including *Anopheles aquasalis*, the primary malaria vector of coastal Central and South Americas and the Caribbean Islands [2].

The biological characteristics influenced by variations in the ability of *Anopheles* vectors to transmit *Plasmodium* (e.g. molecular components of the immune response, microbiota and intestinal physiology) are well studied and have been characterized in established African and Asian models such as *An. gambiae s. s.* and *An. stephensi* [3–5]. The defence mechanisms are critical for establishing the *Plasmodium* in the vectors, and three-quarters of the total ingested parasites die inside the midgut at the start of their life cycle [6]. Changes in the expression of immunity genes occur and vary according to the invaded organ. Also, in this phenomenon the participation of the immune components changes depending on the anopheline species and the malaria parasite [5,7,8].

Several families of innate immune genes control the mosquito response to pathogens. They can influence vector competence, allowing or preventing the development of *Plasmodium*'s life cycle in the mosquito [9–11]. Also, other components of the humoral immune response in mosquito vectors are effector molecules, recognition molecules of microorganisms, signalling pathways and protease cascades. The hemocytes present in the hemolymph are the primary cellular immune response. They participate in phagocytosis, encapsulation, melanization and production of antagonistic molecules to the pathogens [12,13].

It is assumed that the vector's immune response is predominant in controlling *Plasmodium* infection and transmission through the synthesis of anti-pathogen molecules, including those expressed after activation of immune signalling pathways. Several components of the IMD (immune deficiency) and TOLL signalling pathways influence the development of murine and human *Plasmodium* in different anopheline species such as *An. gambiae s. s.*, *An. stephensi and An. albimanus* [14,15]. For example, the silencing of Caspar, the negative regulator of the REL2 in the IMD pathway, had a marked effect leading to increased production of antispasmodic molecules, such as thioester-containing protein-1 (TEP1), leucine-rich repeat protein-7 (LRR7), *Anopheles Plasmodium*-responsive Leucine-Rich Repeat-2 (APL2), and fibrillin-9 (FBN9) [14,15]. Consequently, the suppression of the action of Caspar leads to the continuous activation of the REL2 transcription factor and, therefore, to the increase in the expression of effectors and other molecules regulated by the IMD pathway [14,15]. Based on the findings made in the IMD pathway, the genetic manipulation of *An. stephensi* for the continuous expression of REL2 was carried out, allowing the generation of mosquitoes highly resistant to *P. falciparum* infection [16].

Likewise, immune mechanisms responsible for microbial homeostasis in the mosquito and molecules related to the structural maintenance of the peritrophic matrix have been described as limiting factors for developing plasmodia [17,18]. In *An. gambiae s. s.* and *An. stephensi* mosquitoes, proteins dual oxidase:peroxidase and NADPH-oxidase domains (DUOX) and heme peroxidase 15 (HPX15) participate in catalysing the cross-linking of the mucin layer that delimits the food bolus avoiding the direct contact of the intestinal epithelium with the gut microbiota. The silencing of HPX15 increased the expression of the nitric oxide synthase (NOS) protein, the main effector regulated by the JAK/STAT pathway, eliminating both *P. berghei* and *P. falciparum* through the TEP1 protein [19–21]. On the other hand, the silencing of DUOX protein, a substrate for HPX15 protein, increases the expression of NOS, decreasing the number of *P. berghei* in *A. gambiae s. s.* [21]. However, the silencing of this protein does not affect the parasite development in *An. stephensi* since it does not affect the NOS expression [22].

In this sense, pathogen recognition components are so important in the sporogony cycle of the malaria parasite within the mosquito that they have become candidates for the production of transmission-blocking vaccines (TBVs) or genetic modification in the anophelines [23,24]. As has been shown, for example, for the fibrinogen-1 related protein (FREP1), its presence is necessary for the ookinetes of murine and human plasmodia. When the parasite interacts with this protein, it manages to overcome the peritrophic matrix and continues its movement toward the epithelium of the intestine medium [24,25]. The interaction between FREP1 and ookinetes has been demonstrated in African and Asian anopheline species [24,25]. In addition, according to bioinformatic analysis, it is a highly conserved member of the anopheles genus. Therefore it is speculated to have the same relevance in several malaria vectors, making it a good candidate for TBV [25].

Studies about the interaction between mosquito vectors and *Plasmodium* highlight the importance of understanding the functioning and participation of distinct genes involved in the immune response process. In the future, this knowledge may help raise mechanisms for malaria control. The exciting issue about these data on genetic modification targets or candidates for transmission-blocking vaccines is their conservation among anophelines [19,22,25]. With the annotation of 16 anopheline genomes, it was possible to verify that roughly 60% of orthologous groups are shared within the genus *Anopheles*. Although, in this genomic comparison, intrinsic evolutionary characteristics of the groups or families of genes related to the immune response in anophelines have been recognized. Phylogenetic comparisons could reveal the existence of agonist or antagonist genes against plasmodia in American anophelines, which were detected in African or Asian anophelines in functional experiments [26].

Nevertheless, most of these results were raised from restricted groups of Old World anophelines, which limits what is known about these genes in the neotropical malaria vectors. In the present study, we used the recently annotated genome of the neotropical mosquito *An. aquasalis* to study the composition of the immune response gene families and to make phylogenetic comparisons among four anopheline species of mosquitoes whose genomic data is available. This work is based on homology comparisons with the genes of the immune response of *An. gambiae s. s.*, *An. albimanus* and *An. darlingi*, in addition to using the neighbour-joining (NJ) methodology to make evolutionary inferences with the immune response components identified in *An. aquasalis.*

## 2. Material and methods

### 2.1. Identification and structural characterization of immune response genes in *Anopheles aquasalis*

The *Anopheles aquasalis* mosquito comes from the colony established in the laboratory of Medical Entomology at FIOCRUZ-MG. *An. aquasalis* protein sequences were obtained from genome annotation (GCA_002846955.1) [27]. Putative *An. aquasalis* immune response proteins were identified in the genome and annotated proteins by tBLASTn and BLASTp (amino acid identity greater than 40% and e-value < 0.0001) searches against *Anopheles gambiae s. s.* immune response proteins available at the ImmunoDB database (https://www.ezlab.org/#newick-utils, 6 October 2020) [28].

We used amino acid sequences described by Cao *et al*. [29] as models for blast searches for serine-proteases with CLIP domains. Orthology was initially confirmed by the reciprocal best match method. Genes identified in the genome but not in the annotated proteins had their code regions predicted by GeneWise, using the splice site modelled option [30]. The protein domains of *An. aquasalis* were characterized with the Conserved Domains Database (CDD) database or Interproscan online tool [31,32]. Motifs within some relevant protein domains were identified and visualized using MEME, with the discriminate mode option using proteins of the *An. gambiae s. s.* as a control [33]. Other protein characteristics, such as a signal peptide, were searched for with the SignalP and Macoil programs [34,35].

### 2.2. Evolutionary analysis of *An. aquasalis* immune response genes

*An. gambiae s. s.* immune response proteins were retrieved from the ImmunoDB. Protein sequences from the new world mosquitoes *An. darlingi* were also downloaded (Coari AdarC3.8), *An. albimanus* (STECLA AalbS2.6) and from *An.* gambiae *s. s.* (PEST PEPTIDES AgamP4.12) from the VectorBase database website (https://vectorbase.org/) [36]. To carry out the phylogenetic analysis of the stat1 and stat2 genes, the reference sequences deposited in the Vectorbase database of 14 anopheline species were used (101). Multiple alignments of the sequences between the proteins of the four mosquito species were performed with the Muscle tool with the UPGMA clustering method, with gap opening and gap extension penalties of −2.9 and 0, respectively [37]. The phylogenetic trees were constructed using the NJ method, in which the distance was estimated by the p-distance method implemented in the MEGA-X program. The pairwise deletion option was applied in the NJ tree construction, and the tree topology's accuracy was evaluated using 1000 bootstrap replicates of the sequence alignment [38]. Finally, gene expansion was visualized through a heatmap using the online tool Heatmapper. Six species of mosquitoes were used, including *Culex quinquefasciatus* as a species within the Culicidae family, little related to the *Anopheles* genus, and the *An. stephensi* mosquito, a species of the *Cellia* subgenus. Thus, the mean of each family of immune response genes was calculated from the information of the six mosquitoes and the loss or gain of genes was expressed with the standard deviation for each species [39].

## 3. Results

The data used in this study come from the annotation of the *An. aquasalis* genome [27]. The assembled *An. aquasalis* genome (BioProject PRJNA389759) has 162 944 Mb, distributed in 16 504 scaffolds (N50 14 431) and according to BUSCO analysis, has a completeness of 96.2% of complete single-copy genes, 0.04% complete and duplicated, 2.3% fragmented and 1.5% missing genes. A total of 12 446 protein-coding genes were predicted in the genome and used for the characterization of the components of the immune response.

**Figure 1.**
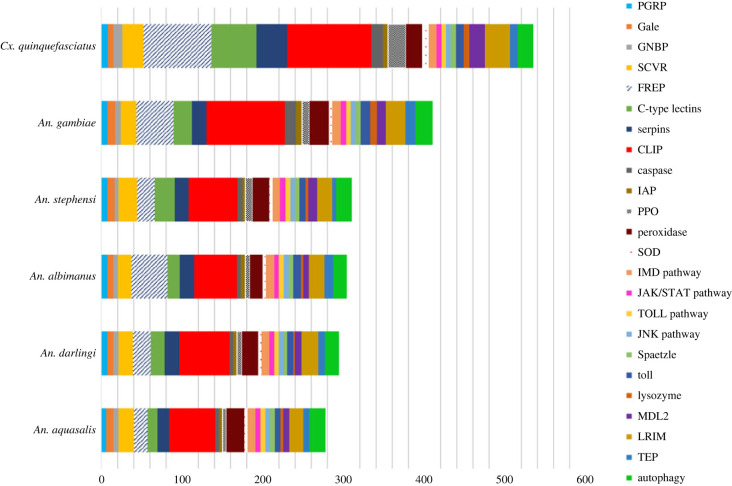
The number of immune response genes in *Anopheles aquasalis* and other anopheline mosquitoes (Culicidae: subfamily Anophelinae). *Culex quinquefasciatus* (Culicidae: subfamily Culicinae) was used as an external group. Each colour in the graph represents an immunity-related gene family.

Two hundred and seventy-eight proteins were identified as of putative immune response function in the *An. aquasalis* genome (figure 1; electronic supplementary material, excel table). These proteins were categorized into 24 families or signalling pathways. Among the sequences, nine genes showed fragmented structures and were manually annotated with the GeneWise program (electronic supplementary material, table S1). Our analysis suggests that *An. aquasalis* maintained a similar quantity of immune genes compared with the other New World mosquitoes (figure 1; electronic supplementary material, table S1) and to *An*. *stephensi* (310 genes), unlike *An.gambiae s. s*., which had approximately 130 more immune response genes. We observed that such differences compared to the *An. gambiae s. s.* is concentrated in two gene families, fibrinogen-related protein (FREP) and CLIP-domain serine protease (CLIP). While *An. gambiae s. s.* has 46 FREP proteins, we found only 17 in *An. aquasalis*. For CLIP proteins, *An. gambiae s. s.* has 97 annotated proteins, while *An. aquasalis* has 57 such genes.

**Figure 2.**
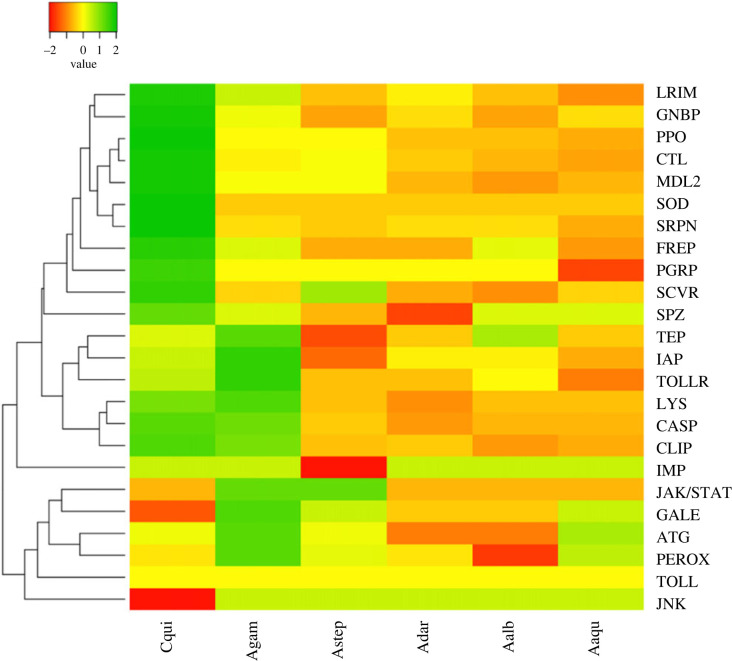
Heatmap of gene copy number variation of immunity-related gene families in *Anopheles aquasalis* and other anopheline mosquitoes (Culicidae: subfamily Anophelinae). *Culex quinquefasciatus* (Culicidae: subfamily Culicinae) was used as an external group. Twenty-four gene families were shown. A phylogenetic tree was generated to represent the gene families and the mosquitoes. The standard deviation represented the specific variation of each family of the immune response by species. Thus, values close to two represent copy gain (Green), and values relative to −2, on the contrary, indicate copy loss (brown) and no variation in those values close to zero (yellow). *Culex quinquefasciatus* (Cqui), *An. gambiae s. s.* (Agam), *An. aquasalis* (Aaqu), *An. darlingi* (Adar) and *An. albimanus* (Aalb).

To facilitate the visualization of gene number variation in each immune-related gene family, we performed a heatmap analysis (figure 2). The heatmap shows the expansion and contraction of gene families in the standard deviation fold. The standard deviation represented the specific variation of each family of the immune response by species. Many gene families present a significant variation in gene copy number in anophelines, remarkably FREP (22.0 copies ± 13.9), CLIP (61.0 ± 17.6), and C-type lectins (17.0 ± 5.5). On the other hand, we observed slight variation in peptidoglycan recognition proteins (PGRPs) (7.0 ± 0.5), Serpins (18.0 ± 1.3) and the IMD pathway (10.0 ± 0.5), and no variation in superoxide dismutase (4 in all anophelines), Toll pathway and JNK pathway genes (both with six genes each). Using *Culex quinquefasciatus* as an external group, we can observe groups of expansions in *An. gambiae s. s.* (first: TEP, apoptosis inhibitors, Toll, lysozyme, caspase and CLIP; second: JAK/STAT pathway, galectins, autophagy and peroxidases). In New-World anophelines, we observe contractions in leucine-rich immune protein (LRIM), gram-negative binding proteins, prophenoloxidases, C-type lectins and Niemann-Pick C2 proteins (MLD2) compared to *An. gambiae s. s.* and *Cx. quinquefasciatus*. Regarding specifically *An. aquasalis*, our attention is drawn to the loss of peptidoglycan recognition protein, serpins and LRIM proteins, as much as the gain of peroxidase and autophagy-related proteins. In the following sections, we will focus on the results of the classic and conserved pathways and those gene families that showed remarkable gains or losses in *An. aquasalis*.

### 3.1. Intracellular signal pathways (IMD, Toll, JAK/STAT and JNK)

**Figure 3.**
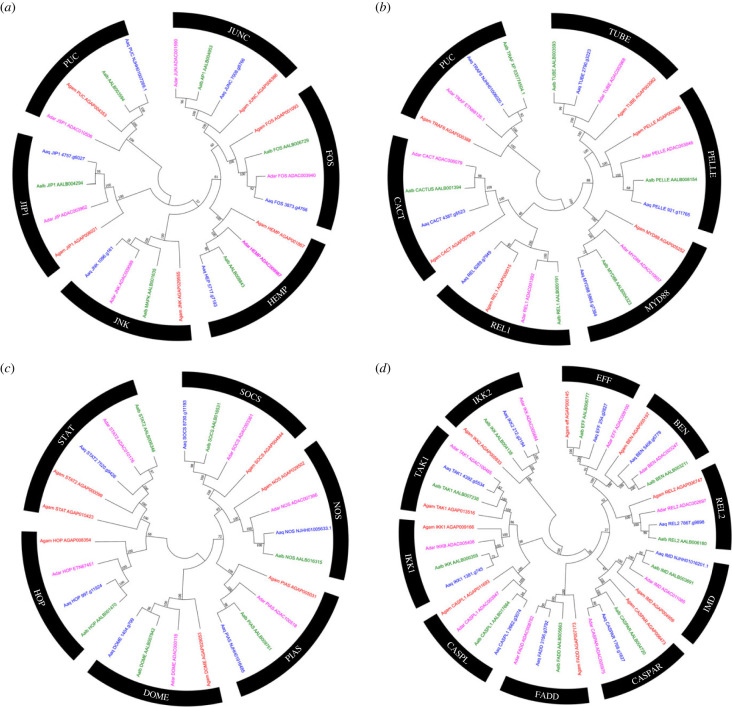
Evolutionary relationships of classical gene families of the IMD, Toll, JNK, and JAK/STAT immune pathways in *Anopheles aquasalis*. (*a*). JNK pathway tree, (*b*). Toll pathway tree, (*c*). JAK/STAT pathway tree, (*d*). IMD pathway tree. Neighbour-joining tree of classical immune systems pathways from the amino acid sequences of the mosquitoes *An. aquasalis* (blue), *An. darlingi* (pink), *An. albimanus* (green) and *An. gambiae s. s*. (red). Bootstrap values were calculated with 1000 replicates, and their values are presented on each tree branch.

After identifying microorganisms and other foreign organisms, the signalling pathways lead to the transcriptional activation of antagonist molecules. The most studied signalling transduction pathways in anophelines are Toll, IMD, and JAK/STAT, but some works also implicate the JNK pathway in control against some species of *Plasmodium* [40–42]. Most genes are part of the signalling pathways identified in *An. gambiae s. s.* are present in *An. aquasalis*, *An. darlingi* and *An. albimanus*, here an orthologous relationship between each individual member of the family was checked (figure 3).

**Figure 4.**
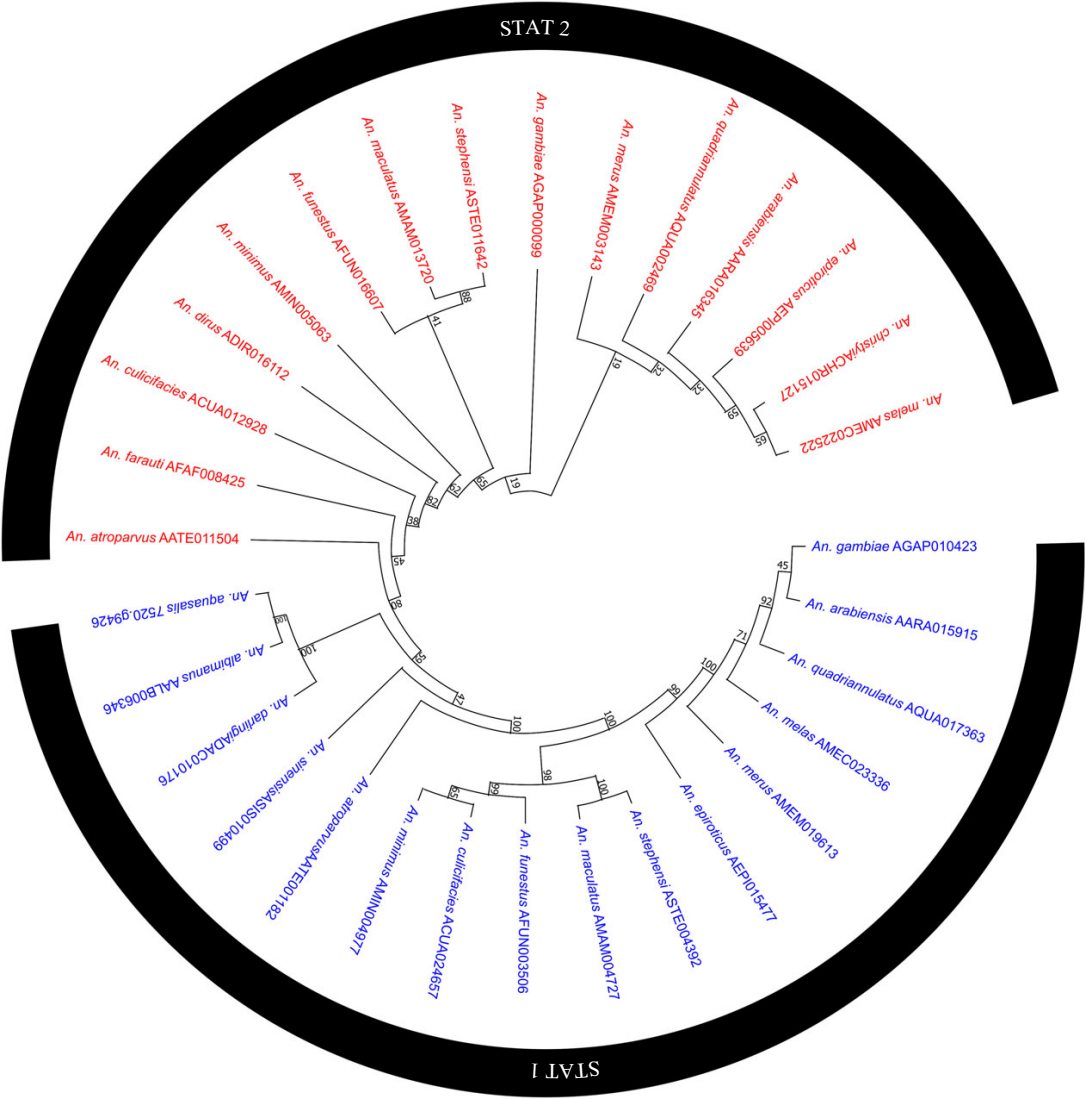
Phylogenetic analysis of the STAT1 and STAT2 genes, carried out with the sequences of 18 anophelines. Bootstrap values were calculated with 1000 replicates, and their values ​​are presented on each tree branch.

Among the exciting events to highlight was the duplication of the STAT gene (JAK/STAT pathway signalling transcription factor) in *An. gambiae s. s.* Apparently, after the divergence between the members of the subgenus *Nyssorhynchus* (American anophelines) and the subgenus *Cellia* (*An. gambiae s. s.*), a new copy emerged for the African mosquito. A result that was also observed when carrying out the phylogenetic tree with the sequences of 18, STAT2 arises in several of the species of the *Cellia* subgenus and even in representatives of the *Anopheles* subgenus. In addition, the absence of STAT2 was not exclusive to American anophelines; the Asian species *An. sinensis*, does not have a copy of this gene (figure 4). In *An. sinensis*, this seems to be the product of a loss, since its congeners, *An. stephensi* and *An. atroparvus*, possess a copy of STAT2. On the other hand, in the STAT2 clade, the orthologous relationship was not very clear for *An. minimus*, *An. dirus*, *An. culicifacies*, *An. farauti* and *An. atroparvus*, even though their sequences had identity values ​​higher than 78% (electronic supplementary material, table S6).

In the JNK signalling pathway (figure 3*c*), *An. aquasalis* and *An. albimanus* had orthologues of the well-known JNK cascade proteins shared with *An. gambiae s. s.,* but *An. darlingi* seems to have lost the PUCKERED gene, a negative regulator of this pathway. It is possible that, *in An. darlingi*, the PUCKERED gene has its role performed by the dual phosphatase gene since both genes have the same regulatory role in insects such as the fruit fly *D. melanogaster* [43]. Furthermore, the genes of all the *An. aquasalis* signaling pathways share identity values with *An. gambiae s. s*. above 41%. (electronic supplementary material, table S2). Within the *An. aquasalis* annotation, the predictions of the IMD genes, the transcription factor regulator PIAS, TRAF6 (Toll pathway signalling TNF Receptor-Associated Factor6), and the NOS genes were fragmented over several scaffolds or missing. Therefore, their predictions were made manually.

### 3.2. Serine protease inhibitors

A total of 15 genes of the serine protease inhibitor family (serpins – SRPN) were found within the genome of *An. aquasalis,* different from the 18 genes identified in *An. gambiae* s. s. The loss of genes was manually reviewed. The SRPN7 and SRPN13 genes from *An. gambiae s. s.* had a low identity value (26% and 48%, respectively) in the BLAST alignment against the *An. aquasalis* genome. Furthermore, this comparison revealed a low score for SRPN7 (102) and low coverage for SRPN13 (12%), making it impossible to annotate these genes manually.

**Figure 5.**
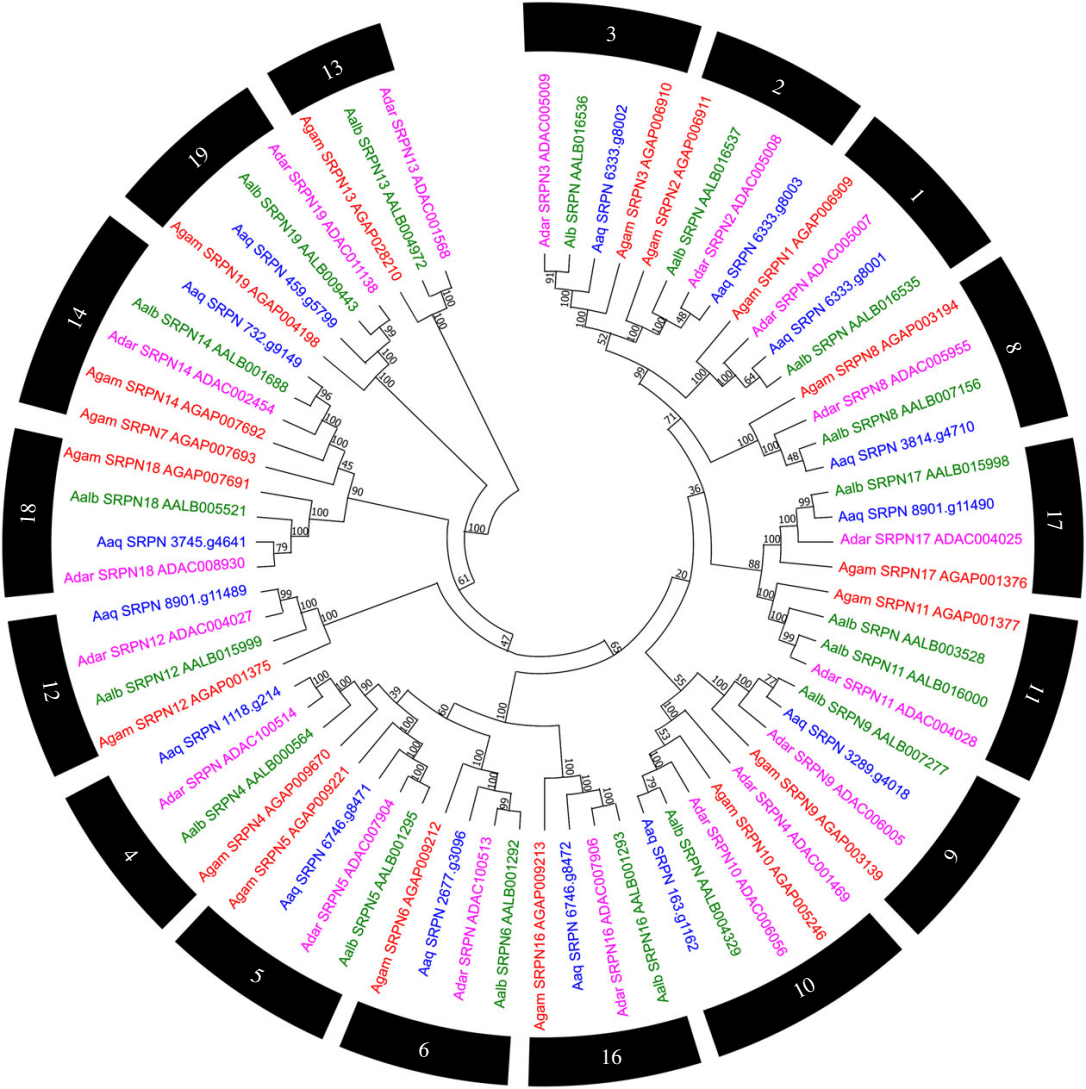
Evolutionary relationship of SERPINs of *Anopheles aquasalis*. Neighbour-joining tree of the family of serine protease inhibitors from the amino acid sequences of the mosquitoes *An. aquasalis* (blue), *An. darlingi* (pink), *An. albimanus* (green) and *An. gambiae s. s.* (red). Bootstrap values were calculated with 1000 replicates, and their values are presented in each tree branch.

In general, a majority of the serpin genes have single-copy orthologues among the anophelines compared. In clade 11, the expansion of *An. albimanus* serpins was identified. Most *An. aquasalis* sequences have values above 52% of identity with *An. gambiae s. s.* (electronic supplementary material, table S2) (figure 5).

### 3.3. Superoxide dismutase proteins

**Figure 6.**
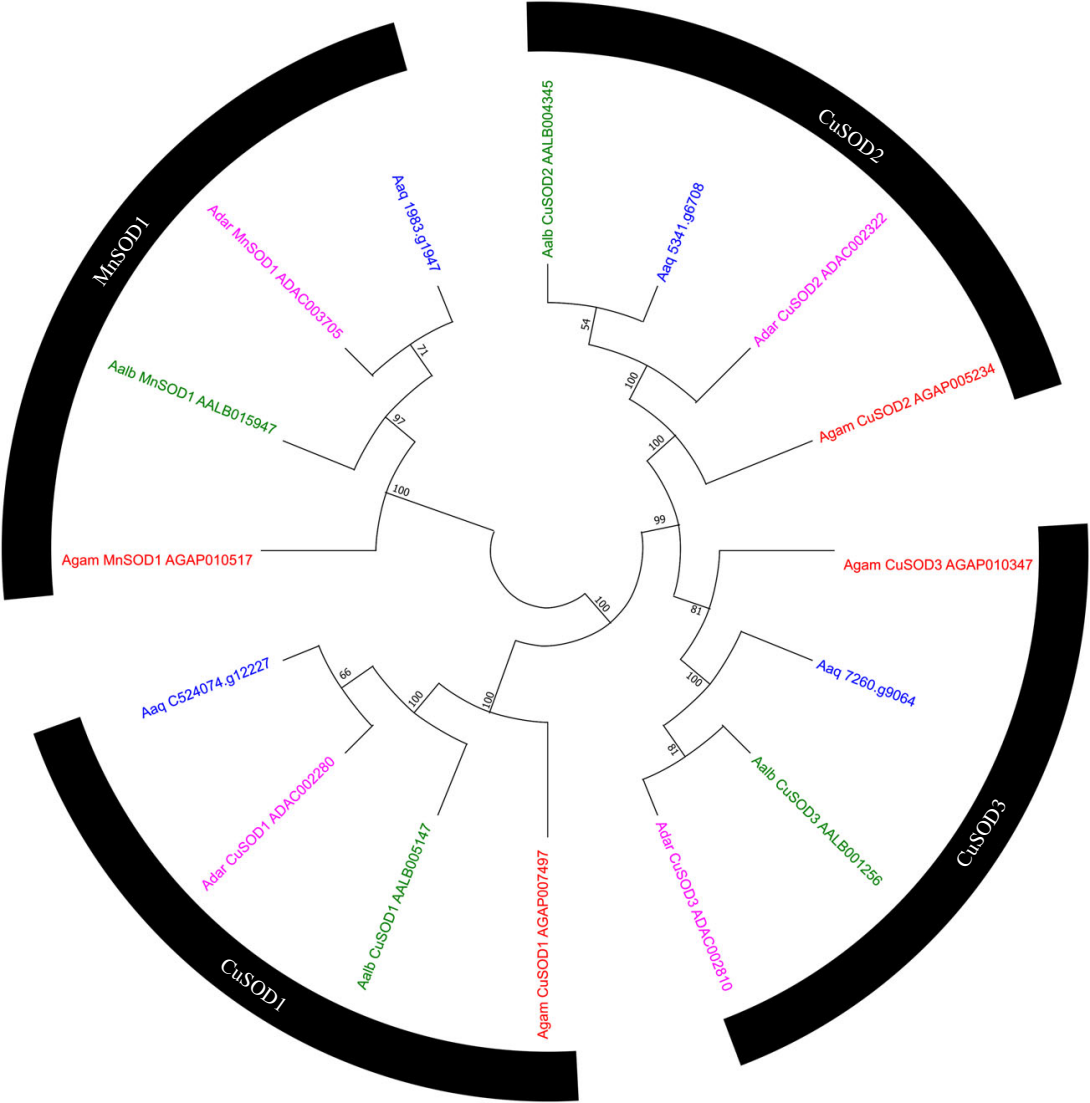
Evolutionary relationship of SODs of *Anopheles aquasalis*. Neighbour-joining tree of the family of superoxide dismutase family from the amino acid sequences of the mosquitoes *An. aquasalis* (blue), *An. darlingi* (pink), *An. albimanus* (green) and *An. gambiae s. s.* (red). Bootstrap values were calculated with 1000 replicates, and their values are presented in each tree branch.

Besides the proteins from the signalling pathways, superoxide dismutase (SOD) was another group of highly conserved genes in the anophelines. Four genes of this family were identified in all mosquitoes with one-to-one orthologues (figure 6), with highly conserved amino acid identity sequence (greater than 90%, Electronic supplementary material, table S2).

### 3.4. Peptidoglycan recognition proteins

**Figure 7.**
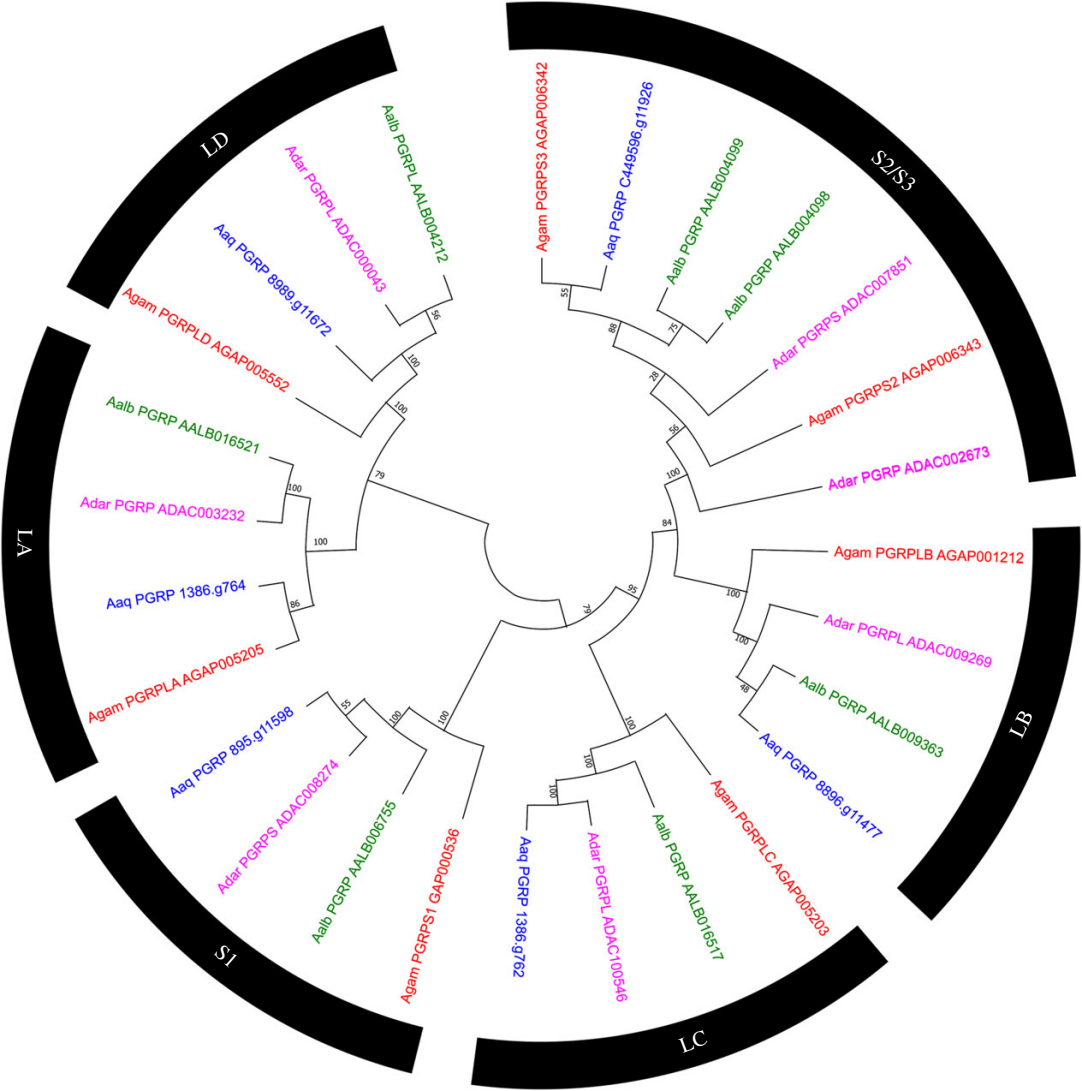
Evolutionary relationship of peptidoglycan recognition proteins of *Anopheles aquasalis.* Neighbour-joining tree of PGRP receptors made with mosquito amino acid sequences*. An aquasalis* (Blue), *An. darlingi* (Pink), *An. albimanus* (Green) and *An. gambiae s. s.* (Red). Bootstrap values were calculated with 1000 replicates, and their values are presented in each tree branch.

In *An. aquasalis* six PGRP were identified, a result similar to the finding in other anophelines, where the composition of this family is from five to seven genes [26,44]. Three sequences of *An. aquasalis* had identity values above 75% compared to their orthologues in the mosquito *An. gambiae s. s.* and at least the identity values are above 59.8%; in this group, the orthologue of AgamPGRPS2 in *An. aquasalis* was fragmented across several scaffolds and was manually annotated. Despite having many single-copy orthologous groups among the four species (LD/LA/S1/LB Groups), in the S2/S3 group, it was impossible to identify orthologues with the AgamPGRPS2 and AgamPGRPS3 proteins for American mosquitoes (figure 7).

### 3.5. Proteins with leucine-rich repeats of the immune response

**Figure 8.**
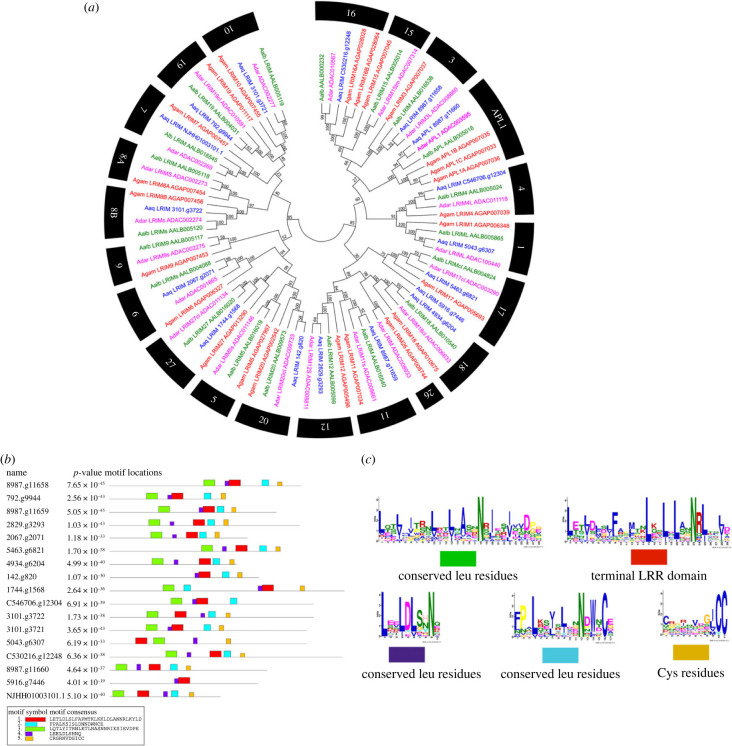
Analysis of leucine-rich repeats of immune response (LRIM) of *Anopheles aquasalis.* (*a*) Evolutionary relationships of anopheline LRIMs. Neighbour-joining tree of LRIM proteins made with *An. aquasalis* amino acid sequences (Blue), *An. darlingi* (Pink), *An. albimanus* (Green) and *An. gambiae s. s.* (Red). Bootstrap values were calculated with 1000 replicates, and their values were presented in each tree branch. (*b*) Distribution of motifs in the LRIM proteins of the *An. aquasalis* mosquito. (*c*) Composition of the motifs of the LRIM proteins in the *An. aquasalis* mosquito.

The proteins with leucine-rich repeats of the immune response (LRIM) are abundant in anophelines; in *An. aquasalis* 17 LRIM genes were described (figure 8*a*). In general, there were orthologues one by one in several of the groups identified. However, evolutionary analysis suggests the loss of LRIM-5, 8A, 9, 15 and 26 in *An. aquasalis*. *An. gambiae s. s.* was the species that had the most gene expansion in this family, mainly in the subgroup of long proteins (figure 8*a*).

The LRIM family has been subdivided into four subgroups according to their structural characteristics and previous evolutionary analyzes. They are recognized as lengthy, short, without coiled-coil and transmembrane [45,46]. The phylogenetic analysis of the present work indicates the exact origin of the short LRIM and those without coiled-coil, despite the structural differences between the two subfamilies (figure 8*b*,*c*; electronic supplementary material, table S1). It was common to find proteins from the two subfamilies between the tree clades without differentiating between the two groups.

Also, the proteins with transmembrane regions (with coiled-coil domains) were represented by groups 15 and 16 in anophelines. In *An. aquasalis*, only one member of these proteins with transmembrane parts was identified (group 16). The other anophelines had a more significant number of copies of them, as evidenced in *An. gambiae s. s.*. These proteins are closely related to the subfamily of long LRIM, with both having a common origin. The long proteins were represented by three groups, composed chiefly of single-copy orthologues for the four anopheline species for two of their genes and the expansion of the APL1 group in *An. gambiae s. s.* and one copy too much in *An. aquasalis* was evident (figure 8*a*).

As pointed out previously, in the LRIM proteins, the leucine-rich repeats (LRR) domain has a particular distribution pattern of residues essential for identifying some proteins of this group with poor identity values [46,47]. In this work, the different motifs with the leucine repeats were determined as described in green, blue, and purple colours; the motif with reproductions of Leu, Trp, and a well-preserved Cys at the end of the LRR domain is represented in red; and the motif with cysteines between the LRR and the coiled coils are represented in yellow (figure 8*b*). Likewise, the distribution of each motif can be observed in figure 7*c*, highlighting that most of the Leu repeat motifs were before the red and yellow motifs, with the conserved residues of Trp and Cys delimiting the LRR domain.

### 3.6. Proteins with fibrinogen domains (FREP)

**Figure 9.**
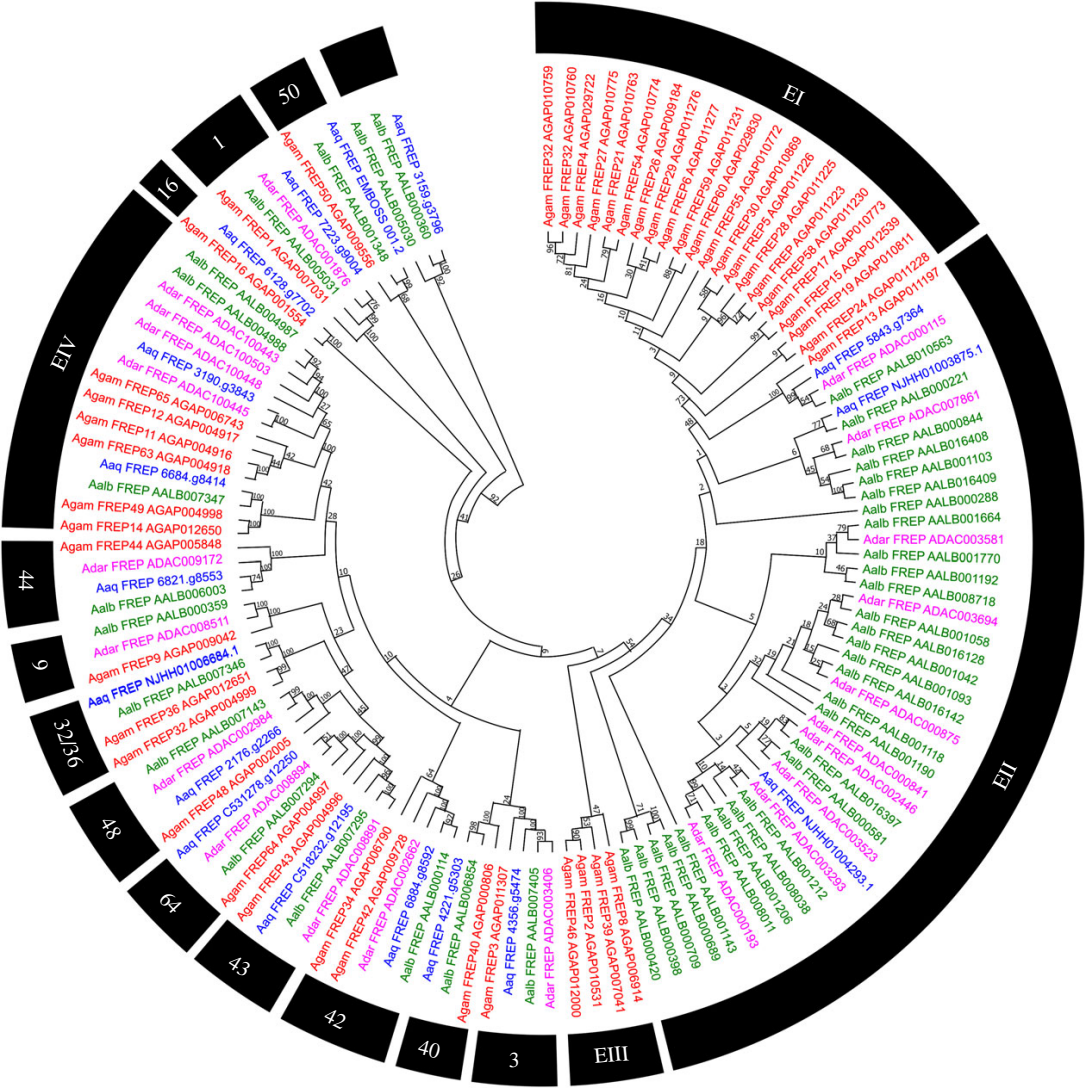
Neighbour-joining tree of proteins with fibrinogen domains (FREP) from the amino acid sequences of *Anopheles aquasalis*. *An. aquasalis* (blue), *An. darlingi* (pink), *An. albimanus* (green) and *An. gambiae s. s.* (red) mosquitoes. Bootstrap values were calculated with 1000 replicates, and their values were presented in each tree branch.

The fibrinogen domain proteins (FREP) genes have heterogeneous abundance in anophelines. In *An. gambiae s. s.,* up to 46 genes have been identified, and the mosquito *An. aquasalis* has 17 FREP proteins. Our evolutionary analysis showed four significant expansions in the FREP family in anophelines. The expansions EI and EIII are exclusive of *An. gambiae s. s.* (figure 9). Conversely, the expansion EII excludes *An. albimanus*, with no apparent orthologues, shared with *An. gambiae s. s.* Concerning *An. aquasalis*, there are only 2 FREP genes in EII, suggesting the loss of some genes and evidencing that many independent expansions occurred in *An. albimanus*. We also observed a fourth event of other additions, EIV, which was more prominent in *An. gambiae s. s.* and *An. darlingi* compared to the other anophelines.

## 4. Discussion

Conservation of proteins at the amino acid and copy number levels is frequently associated with essential genes for the organism's survival and fitness, considering the classical pathways. At first glance, it is possible to observe that the most conserved gene families (concerning copy numbers) were the immune pathways (IMD, JAK/STAT, TOLL and JNK), with 1 : 1 orthologues in almost all anophelines. Proteins of the IMD, JAK/STAT, TOLL and JNK pathways are among the most studied proteins in anophelines. Their importance in the process of vector/parasite interaction has been demonstrated in many insect vectors. The disruption of any of these main pathways by gene knockout or silencing leads to an inability of the vector to defend against pathogens. Interestingly, in anopheline species, duplication in the STAT transcription factor of the JAK/STAT signalling pathway is recognized for some of the members of the *Cellia* and *Anopheles* subgenera [48,49]. Our data analysis suggests that the additional STAT copy emerged after the New and Old World *Anopheles* divergence.

Another highly conserved family of proteins identified here relates to the metabolism of reactive oxygen species (ROS) products, as in the case of superoxide dismutase proteins (SOD). This group is universally present and is believed to have arisen long ago in prokaryotes from increased oxygen production in the atmosphere [50,51]. In *An. aquasalis* and *An. gambiae s. s.*, SOD3 isoforms have emerged to regulate ROS at specific times and in conditions to combat pathogens in the midgut during blood digestion [52,53]. The emergence of SOD3 isoforms in anophelines became a rapid evolutionary strategy to respond to the increase in free radicals at different times or to act in specific tissues during blood feeding of the female, where under certain circumstances it is exposed to pathogens [52,54].

By way of the conserved orthologues between *An. aquasalis* and *An. gambiae s. s.*, we observed that some gene families (Autophagy, Galectins and Peroxidases) have similar numbers of genes (in a 1 : 1 orthologous relationships). Among the American anophelines, the only species with the same number of autophagy proteins of *An. gambiae s. s.* was *An. aquasalis*. The only exception was the ATG12 gene, which was found in two copies in the *An. gambiae s. s.* genome [55], while the three Neotropical mosquitoes (*An. aquasalis*, *An. darlingi* and *An. albimanus*) had only one copy of these genes.

Several components responsible for regulating the catabolic process of autophagy have been conserved in several phyla of eukaryotes such as plants, worms, arthropods and mammals [56–58]. Even so, in these organisms, differences in the number of copies have been found in regulating autophagy's induction and nucleation. In addition, the presence of isoforms has been recorded in the autophagy-related proteins (ATG) [56]. In insects, for example, one of the ATG1 isoforms participates in the activation of autophagy under conditions of starvation or excess food, as well as during metamorphosis, by being phosphorylated in the presence of juvenile growth hormone 20E [59]. Even so, the presence of isoforms does not always lead to phenotypic effects, as was found in *Arabidopsis thaliana* for the ATG12 isoforms and represents a clear example of sub-functionalization. As demonstrated in *A. thaliana*, where in long periods of starvation and darkness, the silencing of both ATG12B and ATG12A genes resulted in increased mortality. According to the results, any of the two ATG12 is sufficient for the association of ATG8 with autophagic bodies. In addition, its expression changes during the life cycle of the plant, with ATG12A frequently expressed in old plants and ATG12B in young *A. thaliana* tissues [60].

ATG8 was one of the proteins conserved in all anophelines except for *An. darlingi*. This protein participates in the final phase of autophagosome formation. In its absence, there is no direction for constructing the pre-autophagosome structure [57,58]. Therefore, ATG8 is ubiquitous in eukaryotes, and its lack in *An. darlingi* may be due to the existence of another protein that can activate autophagosome formation, or it is necessary to annotate ATG8 in this mosquito [56].

Interestingly, other proteins, such as caspases and apoptosis inhibitors related to apoptosis processes, have expanded copy numbers in *An. gambiae s. s.*. The emergence of extra copies in *An. gambiae s. s.* may be related to a greater capacity for regulating the apoptosis process [61–63]. Regarding the galectins, these proteins had more than half of the groups with single-copy orthologues. There was a loss of orthologues in American mosquitoes in two groups and a duplication in *An. gambiae s. s.*. Previously it was shown that galectins underwent an expansion process almost exclusive to mosquitoes, suggesting an influence of hematophagy and exposure to different pathogens for the diversification of this type of recognition receptors in culicids [64,65].

In *An. gambiae s. s.* galectins, there are data on their participation in infections made with Gram-positive bacteria and with *P. berghei*, GALE6–8 also increased its expression during exposure to the alphavirus ONNV, GALE8 being an important antagonist of the virus, here these galectins are well related in a clade in which *An. gambiae s. s.* exhibited a duplication precisely with GALE8/9 [3,66]. Finally, concerning the peroxidases, it is known that they are related to population control of the gut microbiota, avoiding direct contact with effectors of the immune response or increasing the expression of those with bactericidal properties like the DUOX and HPX2 proteins [18,19,22,67]. Relevant heme-peroxidases [19,20], such as HPX15, were found in all anophelines but, again, *An. aquasalis* presented a higher number of 1 : 1 orthologues to *An. gambiae s. s.* than other American anophelines. In summary, by simple parsimony, it is possible to conjecture that *An. aquasalis* and *An. gambiae s. s.* present a conserved ancestral catalogue of genes, with unexpected losses in other American anophelines.

Pattern recognition proteins (PRPs) underwent gene gain and loss dynamics ranging from conserved copy number clusters to extensive species-specific diversifications. For example, in the Gram-negative binding protein (GNBP) and PGRP families, conservation was observed for a few orthologues with possible losses in American mosquitoes. Genes identified as PGRPS2 and GNBP2/3 could have implications in limiting the growth of populations of exogenous bacteria functionally recognized in *An. gambiae s. s.* and *Ae. aegytpi* [68,69]. The absence of the PGRPS2 protein could suggest differences in *Plasmodium* control since in *An. gambiae s. s.* PGRPS2 participates in the reduction of *P. falciparum* and *P. berghei* [70]. Some PRP families present removal only for New World anophelines, such as galectins (except for *An. aquasalis*), C-type lectins, MLD2 and scavenger receptors. Interestingly, for the last two, their functions are not limited exclusively to the immune response, and their diversification could be due to each organism's metabolic or sensory needs [71,72]. Also, it has been demonstrated that *An. gambiae s. s.* and *An. stephensi* MLD silencing increases the oocyst number of *P. falciparum* and *P. berghei* in the midgut, reinforcing the hypothesis previously raised that MLD possibly prevents sterol uptake by parasites [73,74].

As in other insects, CTL proteins (C-type lectins with a single CRD domain) have the most significant diversification in anophelines. In other mosquitoes, it is known that these proteins participate in the homeostatic balance of the microbiota, preventing the activation of antimicrobial peptides (AMPs) [75]. The diversification of these proteins may be in response to each mosquito Field species' bacterial community [75]. Several CTLs are necessary factors that allow the entry of viruses such as Japanese encephalitis virus (JEV), Dengue virus (DENV) and West Nile virus (WNV) into the host cells of *Aedes* and *Culex* mosquitoes, and they are specific for each virus or even for serotypes [76–78]. The CTL evolution could have occurred to protect these insects’ commensal bacteria from the immune and digestion mechanisms activated during the blood meal in the midgut, where viruses and other pathogens take advantage of the production of CTLDs to evade the immune response [79]. This agonist capacity of CTLDs in anophelines was initially recognized in *An. gambiae s. s.* with *P. berghei* in the proteins CTL4 and CTLMA2, these two lectins type C protect the malarial ookinetes from melanization [80,81]. However, in the *An. albimanus*, the protective action of these proteins against plasmodia does not exist, and, according to our analysis based on the phylogenetic data, it is suggested that this functional difference is related to the lack of a true orthologue of CTL4 by the American species [82].

The species-specific diversifications were much more abrupt in the PRP of the FREP, TEP, TOLL and LRIM families in the four studied anopheline species, especially in *An. gambiae s. s.*. This phenomenon was highly accentuated in the FREP family, a previously recognized group with rapid divergence and relaxed evolutionary constraints in insect lineages such as Diptera [83]. FREPs are essential in maintaining immune homeostasis and bacteria elimination. They also work synergistically against murine and human *Plasmodium* [23]. TEP is necessary for the pathogen response in mosquitoes and *Drosophila*, serving as opsonization molecules to control microbes and pathogens, triggering lysis, phagocytosis or melanization [84,85]. There were expansions of TEP in *An. gambiae s. s.* concerning proteins related to TEP1, which is associated with controlling the microbiota and the fight against pathogens derived from blood meal [64,86]. Interestingly, the only group related to TEP1 conserved among the anophelines of the New World was TEP4, a protein with antibacterial properties [47]. LRIMs are described as effectors against pathogens in *An. gambiae s. s.*, but not in *An. albimanus* [46,47,87]. In *An. aquasalis*, only one LRR was recognized under the same conditions. However, most of the *An. gambiae s. s.* LRIMs were present in the New World mosquitoes being 17 of 27 clades composed of single-copy orthologues. Characteristically, Toll receptors showed the same division of three subgroups identified in the insect genomic comparison [88]. The diversification of TOLL receptors, mainly in clades 1, 5 and 9, is attractive since proteins in these groups are commonly related to immune response and embryonic development. The most conserved TLRs among anophelines are in clades 6, 7, 10 and 11. TOLL11 has been described as an antagonist of *P. falciparum* in *An. gambiae s. s.* and has orthologues in the other anopheline species [88–90]. In *An. aquasalis* and *An. albimanus*, transcriptomic studies indicate little activity of TOLL receptors against *Plasmodium* after ingesting a blood meal, even if the TOLL signalling pathway is activated [8,14,91]. Thus, in New World mosquitoes, TLRs are more related to other physiological activities or embryonic development than the immune response. More detailed data are needed for this group to better understand their role in American anophelines.

A few authors have asserted that expansions in the families related to the modulation process of the immune response are widespread [29,64,92]. Its modulatory capacity is due to the particular use of combinations of serine proteases and their inhibitors to activate immune response mechanisms and other physiological processes in which it participates [92]. It is speculated how exposure to specific pathogens could influence the organization and diversification of these protein families. Serine proteases with clip domains suffered more significant species-specific expansions/contractions, especially in *An. gambiae s. s.* in subfamilies B and E [29], also observed in *An. darlingi* and *An. albimanus*. In mosquitoes such as *Ae. aegypti* and *An. gambiae s. s.*, it is widely studied how serine protease with CLIP domains actively regulates melanization as a defence mechanism against bacteria, fungi and parasites [81,93–96]. In *An. gambiae s. s.*, non-catalytic CLIPs are recruited with the TEP1/LRIM1/APL1C complex. This process mediates the accumulation of the complement system on the surface of microorganisms [95,96]. Also, it helps to regulate and activate other serine proteases in cascades where a substantial amount of these proteins with redundant functions influences the intensity of the response by acting in a synergy [97,98]. Some are products of duplications, where sub-functionalization or loss of function possibly occurred, contributing to the regulation of cascades [29]. Species-specific expansions could be related to regulating effectors in response to specific pathogens. In *Ae. aegypti*, for example, CLIP29 and CLIP30/31 paralogues activate the TOLL signalling pathway along with the SERPIN2 protein acting against the fungus *Beauveria bassiana* [93]. The differences observed in protein composition that participate in the melanization process result from the need to regulate the proteolytic cascades to react quickly to pathogen infections or increase the microbiota.

The prophenoloxidase (PPO) system is a hemolymph-based complex of enzymes that, when activated, generate peptides and adhesive proteins, mediating many of the defence functions in arthropods. The expansion of PPO in mosquitoes is well-known. There is a discussion that a series of duplication events have led to the current number of proteins in the culicids [64,99,100]. Even so, losses were identified in the New World's anophelines, especially in *An. aquasalis.* The PPO-6 clade is associated with the melanization of plasmodia in refractory strains of *An. gambiae s. s.* and *An. stephensi* [98,99]. However, PPO-3, derived from PPO-6, is known to cause the melanization of *P. berghei* in *An. gambiae s. s.* by the activation of the PGE2 protein. Moreover, in *An. dirus*, PPO4 has an antispasmodic effect independent of the melanization [101,102].

Overall, the results presented in this study indicate a variable pattern of evolution in the immune response genes of the studied anopheline mosquitoes. Environmental factors, such as exposure to different pathogens and differences in the composition of the microbiota, could shape the expression of this group of genes. This study formulated a catalog of the evolution of immune response genes in *An. aquasalis*, the primary malaria vector of coastal Central and South America and the Caribbean Islands. The *An. aquasalis* mosquitoes used in this study are from a well-established colony that has also analysed the interaction process with murine and human malarial *Plasmodium* strains. The results raised here will contribute knowledge of the study of a Neotropical vector, which may open opportunities for future malaria control in the malaria-endemic areas of the New World.

## Data Availability

Data are available upon request from the corresponding author or related data bank cited in the material and methods. Additional information is provided in electronic supplementary material [103].
